# Assessing Vodka Authenticity and Origin in Vietnam's Market: An Analytical Approach Using FTIR and ICP-MS with Multivariate Statistics

**DOI:** 10.1155/2024/5652559

**Published:** 2024-07-16

**Authors:** Minh Truong Ngoc, Quang Trung Nguyen, Van Thinh Pham, Le Tuan Anh Hoang, Viet Anh Le, Van Nhan Le, Ha Minh Duc Tran, Tien Dat Nguyen

**Affiliations:** ^1^ Center for High Technology Research and Development Vietnam Academy of Science and Technology, 18 Hoang Quoc Viet Street, Cau Giay, Hanoi 100000, Vietnam; ^2^ Institute of Environmental Science and Public Health, 18 Hoang Quoc Viet Street, Cau Giay, Hanoi 11353, Vietnam; ^3^ Faculty of Food Science and Technology Ho Chi Minh City University of Industry and Trade, Ho Chi Minh City 70000, Vietnam

## Abstract

Vodka constitutes a significant sector of Vietnam's alcohol industry, including both domestic and imported varieties. However, this diversity faces challenges from illegal imports and adulterated products, threatening consumer health and brand integrity. This study employs Fourier transform infrared spectroscopy (FTIR) and inductively coupled plasma mass spectrometry (ICP-MS) to analyze 300 vodka samples from five brands collected across Hanoi. Significant variations were found in elemental compositions, with sodium concentrations ranging from 205.67 *μ*g/L to 1269.24 *μ*g/L and magnesium levels from 65.57 *μ*g/L to 1453.34 *μ*g/L. Principal Component Analysis (PCA) of the FTIR and ICP-MS data effectively differentiated the samples, with the first two principal components explaining 84.78% and 73.02% of the total variance, respectively. The PCA plots revealed distinct chemical profiles, notably isolating Rocket Vodka. These findings enhance food safety enforcement, protect consumer rights, and preserve brand reputations. The study underscores the importance of advanced analytical tools in combating beverage adulteration, ensuring public health, and maintaining market integrity, offering a replicable model for similar research in other regions.

## 1. Introduction

Food control and provenance determination have numerous advancements by using the fingerprinting technique in food authentication [1–4]. Most of these researches were based on data obtained from analytical techniques and processed with one or multiple multivariate statistical analysis techniques [2, 4, 5]. The samples of interest are first analyzed with a specific analytical technique to achieve a data set which includes mostly quantitative information of elements or compound of interest such as traced element content, isotope ratio, or spectrum of the samples [5–8]. Then multivariate statistical analysis method will be applied to study the significant data and categorize the samples based on these signature differences [9, 10]. The methods rely on the assumption that certain components of the production conditions and environment will reflect on the chemical composition of the product [5–8].

Vodka is a distilled alcoholic beverage, originating in Poland and Russia, consisting mainly of water and ethanol (37.5–55% alcoholic content) [11]. This alcohol is the product of ethyl alcohol of agricultural origin such as the fermentation of potatoes, grains, or other agricultural products [12, 13]. The characteristic of vodka comes from multiple filtering through activated charcoal, diluting with water, distilled, and demineralized [14–16]. Traditionally, vodka is clear, colorless, with no aroma or taste, and the Russians called it voda for “water” [17]. However, most brands in the vodka market now have both traditional and flavor vodka. The changes in the environment have brought changes to the distillery industry and the production of some type of vodka [13, 18]. Furthermore, with the standard properties of being clear, colorless, with no aroma and taste, an easy production method with wide range of base material, and the price range higher than beer, vodka becomes the target for illicit products [13, 19, 20]. The variety of vodka brands and types available on the market has become the perfect environment for adulterated and counterfeit vodka to thrive.

Drinking alcohol in Vietnam is widely considered a social etiquette, a way to build and maintain social networking, in both workplace and household etiquette. Also, Vietnamese consume alcoholic beverages for any celebration [21]. Hence, Vietnam was ranked 16^th^ among the largest alcohol-consuming countries in 2016, ranked 2^nd^ in South East Asia, and 3^rd^ in Asia in 2022 [21, 22]. The high consumption rate leads to high demand for products, and illicit products started to infiltrate the alcohol market. According to Trend Economy, Vietnam's alcohol export in 2020 was up to 50% of total beverages export (over 121 million USD) [23]. Currently, in Vietnam, many counterfeit products are being sold widely on the market; this status does not make an adverse impact on the health of consumers but also on the image, trademark, and economy of the manufacturer, causing loss on sales. Consuming adulterated/illicit alcohol can cause alcohol poisoning that leads to liver failure, kidney failure, blindness, coma, and eventually death; patients who survive alcohol poisoning can have hepatitis, gastrointestinal bleeding, cardiovascular disease, and high blood pressure [20, 24, 25].

There are reports of discriminating food products using methods such as gas chromatography (GC) and high performance liquid chromatography (HPLC) [15, 26]. However, such methods require complicated optimization and costly reference material [15, 26]. On the other hand, Fourier transform infrared-attenuated total reflectance (FTIR-ATR) and inductively coupled plasma–-mass spectroscopy (ICP-MS) are quick and effective methods, especially for liquid samples [4, 27–29]. Therefore, this research aims to identify different vodka products available in Vietnam's market using ICP-MS and FTIR results combined with multivariate statistics.

Fourier transform infrared (FTIR) spectroscopy is based on the differences in the structure of each molecule and they absorb a different amount of energy from the infrared (IR) source [30, 31]. The covalent bonds within the molecule absorb specific wavelengths and change the vibrational energy in the bond. The vibrational type (stretching or bending) caused by IR radiation depends on the atoms in the bond [30, 31]. Attenuated total reflection (ATR) is a method to directly observe a sample in the solid or liquid state. ATR involves an IR beam that travels from a medium with a high refractive index (ATR crystal) to a low refractive index (sample) and reflects with reduced intensity [32].

Inductively coupled plasma mass spectrometry (ICP-MS) is a multielement analytical technique to determine trace element levels [27, 33]. This method is applied in many different fields such as environment (water, soil, air, sediment, and waste), product testing, quality control, agriculture, and food safety [27].

## 2. Material and Method

### 2.1. Sample Collection and Preparation

Vodka samples from five distinct brands—Vodka Hanoi, Men's Vodka, Aligator Vodka, Hanoi Wine, and Rocket Vodka—were systematically collected from major retail outlets and supermarkets across Hanoi. A total of 300 samples were acquired, with each brand contributing 60 samples. To ensure integrity and prevent contamination, each sample was maintained in a fully sealed condition at ambient room temperature. Furthermore, all samples were meticulously labeled with details including the location of collection, date of processing, and a unique coding number to facilitate precise tracking and identification during analysis.

### 2.2. IR Spectral Acquisition

The spectral acquisition was conducted using the Nicolet iS50 FTIR Spectrometer (Thermo Fisher Scientific, Massachusetts, United States), which is equipped with an integrated attenuated total reflection (ATR) system featuring a zinc selenide (ZnSe) crystal. This setup allows for the measurement of both mid and long infrared regions. For the analysis, 3 to 4 drops of each sample were directly applied onto the ZnSe crystal surface. Each sample underwent 16 scans at a resolution of 4, and the procedure was repeated six times for each sample to verify reliability. A blank measurement was conducted between each sample analysis to ensure accuracy. The spectrometer was meticulously cleaned with ethanol following each measurement session. The spectral data were captured and analyzed using OMNIC software, covering a wavelength range from 4000 to 400 cm^−1^.

### 2.3. Elemental Determination by ICP-MS

#### 2.3.1. Chemicals

Nitric acid (HNO_3_) at a 65% concentration and hydrogen peroxide (H_2_O_2_) at 30% were sourced from Merck, USA. Ultrapure deionized water, possessing a resistivity of 18.2 MΩcm, was produced using the Milli-Q Plus water purification system (Millipore, Bedford, MA, USA). Certified Reference Materials (CRMs) were employed from CPA Chem, which provided a standard solution containing 100 mg/L of multiple elements including Al, Ag, As, B, Ba, Be, Bi, Ca, Cd, Cs, Co, Cr, Cu, Fe, In, K, Li, Mg, Mn, Mo, Na, Ni, Nb, Pb, Rb, Sb, Se, Sr, Ti, Tl, V, U, and Zn, all in a 5% nitric acid solution. This was used to construct the calibration curves. Additionally, a solution of 9 elements at 10 mg/L concentration—Bi, Ho, In, ^6^Li, Lu, Rh, Sc, Tb, and Y, also in a 2% nitric acid solution—was utilized as an internal standard for the analyses.

#### 2.3.2. Sample Preparation and ICP-MS Methods

Each vodka sample, initially 50 mL in volume, was heated at 80°C until reduced to 20 mL. This concentration step primarily aims to remove ethanol, a volatile component that could create hazardous pressures during subsequent microwave digestion processes. After cooling, the concentrated sample was transferred to a 50 mL volumetric flask and diluted to the mark with ultrapure deionized water. For chemical analysis, 5 mL of this prepared solution was pipetted into a Teflon tube. To this, 2 mL of 65% nitric acid (HNO_3_) and 0.25 mL of the internal standard were added. The mixture was allowed to sit overnight to stabilize.

The samples were then subjected to microwave-assisted digestion using a MARS 6 system (CEM, North Carolina, United States) with the following digestion protocol specifically designed for food samples: power set between 1030 and 1800 W, a ramp time of 20–25 minutes, a hold time of 15 minutes at 210°C, followed by a cooling period of 20 minutes. Post-digestion, the digested solution was transferred to a 25 mL volumetric flask and brought up to volume with deionized water. The final step involved filtering the prepared sample into a coded falcon tube, ensuring it was ready for inductively coupled plasma mass spectrometry (ICP-MS) analysis. This procedure ensures the removal of organic contaminants and the stabilization of the sample matrix, facilitating accurate trace elemental analysis.

The experiment uses iCapTQ ICP-MS (Thermo Fisher Scientific, Bremen, Germany) to analyze the elements in the samples. The elements and their most abundance isotopes measured are ^11^B, ^23^Na, ^24^Mg, ^39^K, ^43^Ca, ^48^Ti, ^51^V, ^52^Cr, ^55^Mn, ^57^Fe, ^59^Co, ^60^Ni, ^63^Cu, ^66^Zn, ^98^Mo, ^111^Cd, ^115^In, ^121^Sb, ^138^Ba, ^202^Hg, and ^208^Pb. The samples were diluted appropriately with deionized water and measured with the instrument. The operational parameters for the ICP-MS analysis were as follows: RF power was set to 1200 W, with a sample depth of 5 mm. The plasma gas flow rate was maintained at 15 L/min, the carrier gas flow rate at 1.05 L/min, and the makeup gas flow rate at 0.9 L/min. The spray chamber temperature was controlled at 2°C. For spectral analysis, each spectral peak was measured with 3 points per peak, and each reading consisted of 10 sweeps. The samples were appropriately diluted with deionized water before measurement.

In this study, the instrument detection limits were calculated using the raw intensity data from the standard and the blank (using an ultrapure 2% nitric acid matrix) according to the equation as follows:

(1) $$\begin{array}{c} \text{IDL}=3{\text{SD}}_{\text{Blank}}\times \frac{C_{x}}{\left(S_{x}-S_{\text{Blank}}\right)}, \end{array}$$

in which IDL is the instrument detection limit, SD_Blank_ is the standard deviation of measurement, *C*_*x*_ is the mean signal for the standard, *S*_*x*_ is the signal for *C*_*x*_, and *S*_Blank_: is the signal for blank.

The method of detection limit (MDL) was calculated as

(2) $$\begin{array}{c} \text{MDL}=\text{IDL}\times \frac{v\text{olume}}{{\text{mass}}_{\text{constant}}}. \end{array}$$

### 2.4. Statistical Analysis

Statistical analysis of the collected data was conducted using STATISTICA 12 software (Dell, USA). Principal Component Analysis (PCA) was employed to explore and visualize the differences among vodka brands based on data obtained from both Fourier transform infrared spectroscopy (FTIR) and inductively coupled plasma mass spectrometry (ICP-MS) analyses. The results of this multivariate statistical technique were presented in two forms: a score scatter plot derived from FTIR analysis and a combined score scatter plot with *X* and *R* moving charts from the ICP-MS analysis. These visual representations allow for the identification of clustering patterns and outliers, thus highlighting the variances between the brands based on their spectral and elemental signatures. This analytical approach enhances the understanding of brand-specific characteristics and assists in the authentication process.

## 3. Results and Discussion

### 3.1. Infrared Spectroscopy Analysis

**Figure 1:**
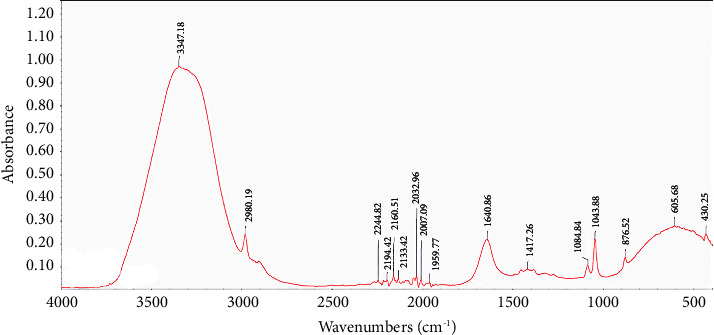
FTIR spectrum of a vodka sample.

Figure 1 illustrates a typical characteristic FTIR spectrum of a vodka sample, displaying various peaks across the spectrum.

Despite initial observations, when the spectra from different brands are superimposed, the distinctions among them are minimal, indicating high similarity across the brands in the spectral data. Given the challenge in discerning differences based solely on the full spectra, a targeted approach was adopted. Specific, significant peaks within the spectrum were carefully selected for deeper analysis. These selected peaks were then analyzed using multivariate statistical analysis, specifically Principal Component Analysis (PCA). This method enabled a more detailed examination of the underlying differences and facilitated the classification of the vodka samples according to brand. This approach underscores the utility of combining targeted spectral analysis with advanced statistical techniques to enhance differentiation and classification in complex sample sets.

In the analysis of vodka samples, specific functional groups' peaks were identified to correlate with the possible classes of compounds present in the beverage, as referenced in the literature [10] (Table 1). Among these, the peak at 3347 cm^−1^, located within the range of 3600−3000 cm^−1^, is attributed to the –OH (hydroxyl) group stretching, a characteristic feature of alcohols and phenols [32]. Another significant band observed at 2980 cm^−1^, falling within the 3000−2500 cm^−1^ range, is associated with the stretching vibrations of –CH groups, indicative of the presence of alkenes, alkynes, and alkanes [32, 33].

Furthermore, a distinct sharp peak at 1640 cm^−1^ is identified as the C=C stretching typically of monosubstituted alkenes [32]. In the spectral region between 1300 and 1000 cm^−1^, notable peaks include one at 1084 cm^−1^, which is consistent with the C–O stretching vibrations found in alcohols. Additionally, a peak at 1043 cm^−1^ suggests the presence of S=O stretching, characteristic of sulfoxide compounds [32]. These identifications aid in the deeper chemical profiling of vodka samples, providing insights into their compositional nuances.

### 3.2. ICP-MS Elemental Measurement

Table 2 presents the elemental composition of vodka from five different brands: Hanoi Wine, Men's Vodka, Aligator Vodka, Vodka Hanoi, and Rocket Vodka.

The analysis reveals the presence of macro-elements such as Na ranging from 205.67 to 1269.24 *μ*g/L, Mg from 65.57 to 1453.34 *μ*g/L, K from 239.33 to 5856.33 *μ*g/L, and Ca from 255.72 to 3157.16 *μ*g/L. The concentrations of these elements vary significantly between the brands, highlighting distinct profiles. However, these macro-elements are also prone to environmental influences due to their ubiquitous presence. For example, elements like Fe and Cu may derive from distillery equipment and processes [34], while Pb can be introduced through contaminated water supplies [35]. Given these factors, the reliability of these elements as indicators of brand distinction or authenticity in vodka might be compromised.

To address this, the Principal Component Analysis (PCA) used for brand differentiation and classification in this study selectively employs a subset of trace elements less likely to be influenced by environmental factors. The elements included in the PCA are Li, Al, Ti, V, Mn, Cr, Co, Ni, Zn, Mo, Cd, Sb, and Ba. These choices enhance the analytical rigor and reliability of the PCA results by focusing on elements with more controlled and specific sources in the production process.

### 3.3. Brand Classification by Principle Component Analysis (PCA)

Principal Component Analysis (PCA) was applied to examine the compositional differences among 300 vodka samples from five brands, effectively visualizing their distinctiveness. The PCA model utilized score scatter plots, which served as a screening tool to display the contributions of the principal components (PCs) to the overall variance observed within the sample dataset. These plots revealed that the selected PCs account for a significant proportion of the total variation, indicating that these components encapsulate the essential information of the variables under study.

The efficacy and reliability of the PCA models are quantitatively assessed using the *R*^2^ and *Q*^2^ values. The *R*^2^ value measures the proportion of variance in the data that is explained by the PCA model, serving as an indicator of the model's fit. A higher *R*^2^ value suggests a model that accurately captures the major trends and differences among the samples. Conversely, the *Q*^2^ value assesses the model's predictive ability based on a cross-validation procedure. It indicates how well the PCA model can predict new data points and is crucial for evaluating the model's generalizability and robustness.

By analyzing these values, researchers can determine the quality of the PCA model and its utility in distinguishing between vodka brands based on their chemical composition. This methodological approach provides a robust framework for identifying brand-specific signatures and ensuring the authenticity of products in the market.

#### 3.3.1. Principal Component Analysis of FTIR Data

**Figure 2:**
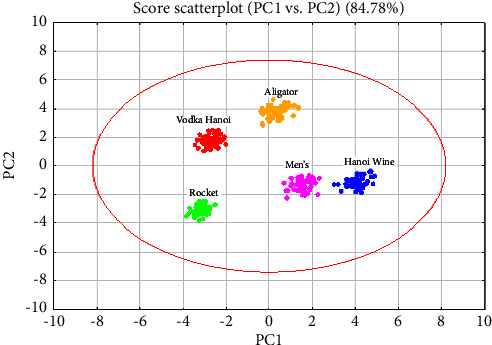
PCA score plot of FTIR analysis of vodka samples.

The outcome of the Principal Component Analysis (PCA) vividly illustrates the compositional distinctiveness among the five vodka brands (Figure 2). The PCA results clearly demarcate each brand, although the distributions for Men's Vodka and Hanoi Wine are relatively proximate to each other. Despite their closeness, a discernible boundary still separates these two brands, suggesting subtle but significant differences in their compositions.

The PCA plot further highlights that Rocket Vodka's results are positioned distinctly in the third quadrant, isolating it from the clusters formed by the other brands. This spatial separation within the PCA plot indicates that Rocket Vodka possesses a unique chemical profile compared to the others, emphasizing the effectiveness of PCA in capturing and visualizing such differences.

This graphical representation serves as a powerful tool for understanding how each brand's specific compositional traits contribute to their placement on the PCA plot, thereby providing insights into the underlying variables that drive brand differentiation in the vodka market. Such analysis not only aids in quality control and brand authentication but also helps in understanding consumer preferences linked to compositional differences.

Table 3 in the PCA analysis of the FTIR data set indicates that the sum of squares of the first four components accounts for 91.28% of the total variation in the samples. Notably, the first two principal components (PCs) contribute significantly, representing 84.78% of this variation. This substantial proportion suggests that these initial two PCs encapsulate the primary information about the variables being analyzed.

The effectiveness of the PCA model is quantified by several key metrics: *R*^2^ and *Q*^2^ values. Specifically, the cumulative *R*^2^*X* (*R*-squared) value for the first two components stands at 84.78%. This *R*^2^*X* value reflects the proportion of variance in the dataset that is successfully explained by PCs 1 and 2, confirming a strong model fit that captures the majority of data variability. Furthermore, the cumulative *R*^2^ (Q-squared) value, which measures the model's predictive ability based on cross-validation, is recorded at 76.05% for the first two components.

The high *Q*^2^ value indicates that the model not only explains but also reliably predicts new data within this variance scope. Together, these values demonstrate that the first two principal components are highly effective in capturing and predicting the essential characteristics of the vodka samples based on FTIR data. This robustness in model quality underscores the utility of PCA in detailed compositional analysis, facilitating insightful distinctions and reliable predictions in complex datasets like those typical in quality control and brand differentiation studies in the beverage industry.

#### 3.3.2. Principal Component Analysis of ICP-MS Data

**Figure 3:**
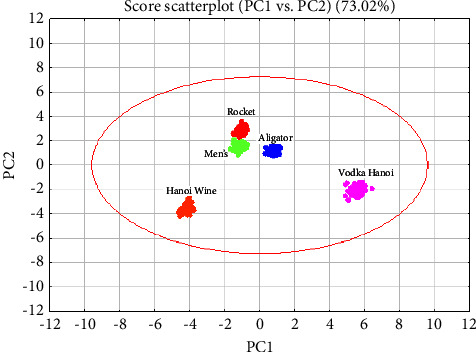
PCA score plot of ICP-MS analysis of vodka samples.

The scatter plot derived from the PCA analysis provides a visual representation of the distribution of vodka samples based on their metal content (Figure 3). Notably, the plot reveals a partial overlap between the samples from Rocket Vodka and Men's Vodka. This overlapping suggests a degree of similarity in the metal composition of these two brands, indicating that they may share certain sourcing or production processes that affect their elemental profiles.

Conversely, the samples from Hanoi Wine and Vodka Hanoi are distinctly separated on the plot, indicating a unique metal content that differentiates them from each other and from the rest of the brands analyzed. This clear demarcation suggests that the metal constituents in Hanoi Wine and Vodka Hanoi are significantly different, which could be due to differences in the raw materials used, the distillation process, or other factors specific to each brand.

Such distinctions in the PCA scatter plot are crucial for identifying and understanding the underlying factors that contribute to the compositional differences among brands. They not only help in the authentication of the vodkas but also in tailoring specific quality control measures for each brand based on their unique metal content profiles.

Table 4 from the PCA analysis on a different dataset reveals that the sum of squares for the first eight principal components (PCs) accounts for 97.51% of the total variation among the vodka samples. Specifically, the first two components make up a substantial portion, contributing 73% to this total variance. This high proportion underscores the significance of these two components in capturing the essential characteristics and variability in the dataset.

The quality of the PCA model is further quantified by the cumulative *R*^2^*X* (*R*-squared) and *Q*^2^ (*Q*-squared) values associated with these components. The *R*^2^*X* value stands at 73.02%, indicating that approximately 73% of the variance in the sample data can be explained by the model constructed using just the first two PCs. This suggests a strong model fit, capturing a large fraction of the information contained in the data.

The cumulative *Q*^2^ value is 67.48%, which measures the predictive accuracy of the model based on a cross-validation method. A *Q*^2^ value of 67.48% implies that about 67% of the dataset's variance can be predicted by the model, highlighting its practical effectiveness in forecasting or simulating similar data. These metrics are essential for evaluating the PCA's capability not only to describe but also to anticipate the behavior of new sample data, which is particularly valuable for process control and quality assurance in vodka production.

**Figure 4:**
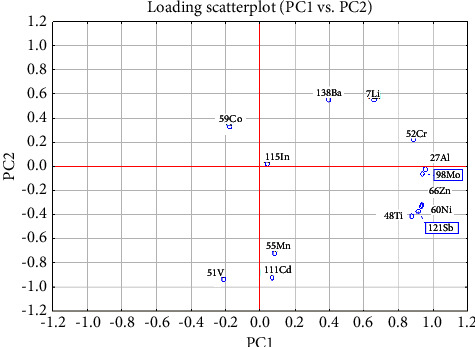
Loading scatter plot for ICP-MS data.

The two-way scatter plot in Figure 4, constructed using the first two principal components (PC1 and PC2), provides a graphical representation of the influence of various elements on the PCA results. In this plot, the position of each element relative to the origin of the coordinate system indicates its level of influence on the analysis. Elements that are positioned further from the origin exert a more significant impact on the PCA model, reflecting their substantial role in differentiating the samples.

According to the plot, elements such as V, Ba, Li, Cr, Al, Mo, Zn, Ni, Sb, and Ti are identified as having a heavy influence on the PCA results. Their strong presence on the plot suggests that these elements are key discriminators among the vodka samples, contributing significantly to the variance captured by the first two principal components.

This visualization is crucial for understanding which variables (in this case, elements) are most critical in the classification and differentiation process within the dataset. By identifying these influential elements, researchers and quality control teams can focus on these specific components during analysis, ensuring more targeted and effective monitoring and verification of the vodka samples. This approach not only enhances the robustness of the PCA model but also aids in pinpointing potential areas for quality improvement or regulatory focus in vodka production.

**Figure 5:**
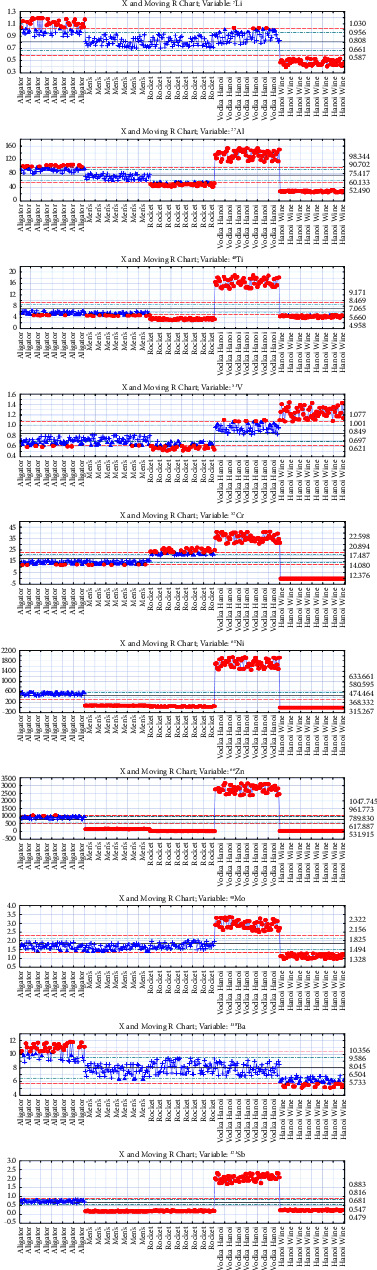
Moving range chart for each element.

The moving range charts provide a statistical depiction of the distribution of metal concentrations among various vodka brands (Figure 5). These charts are useful for visualizing both the central tendency (mean) and variability (range) of metal concentrations within the samples.

From the analysis, it appears that the brands Aligator, Men's, and Rocket Vodka exhibit relatively similar metal concentrations across all cases, suggesting a degree of uniformity in either their production processes or sources of raw materials. Such consistency might imply standardization or shared supply chains among these brands.

In contrast, Vodka Hanoi consistently shows the highest metal concentrations in seven out of the ten charts analyzed. This could indicate different sourcing of ingredients, distinct manufacturing processes, or less rigorous purification steps compared to the other brands. High metal concentrations might affect the taste, quality, and possibly safety of the vodka, depending on the specific metals and their concentrations.

On the other hand, Hanoi Wine's vodka generally has the lowest metal concentrations in nine out of ten charts, except for vanadium, where it exhibits the highest concentration. This overall lower level of metals suggests more effective purification or different sourcing and production practices that minimize metal content, potentially leading to a cleaner and possibly more premium product.

These findings highlight significant differences in metal content between brands, which could be critical for consumer safety, regulatory compliance, and brand positioning in the market. Quality control measures and sourcing practices might need to be reviewed, especially for brands like Vodka Hanoi, to ensure product safety and maintain consumer trust.

## 4. Conclusion

The illicit alcohol trade poses significant risks to human health and the global economy, making the development of robust quality assurance methods for legitimate alcoholic products critically important. Techniques such as multielement fingerprinting via inductively coupled plasma mass spectrometry (ICP-MS) and vibrational spectroscopy using Fourier transform infrared (FTIR) spectroscopy offer rapid and effective means for analyzing the composition of liquid samples.

The application of multivariate statistical methods, particularly Principal Component Analysis (PCA), has proven effective in analyzing and categorizing extensive datasets. In the context of this study, PCA has successfully differentiated 300 vodka samples based on brand distinctions, demonstrating its utility in distinguishing between products with high precision.

This capability highlights the potential to identify unknown vodka samples purely based on their FTIR or ICP-MS data profiles. Such analytical techniques can be integrated into quality assurance protocols to authenticate vodka brands and detect counterfeits, thereby safeguarding consumer health and protecting brand integrity. Furthermore, these methods offer a scalable approach to tracking and monitoring vodka products in the marketplace, ensuring compliance with safety standards and helping to combat the trade in illicit alcohol. This strategic application not only enhances consumer trust but also supports regulatory bodies in enforcing food and beverage safety regulations.

## Figures and Tables

**Table 1:** Corresponding functional groups and possible compound class of FTIR signal of vodka sample.

Wavelength region (cm^−1^)	Selected peaks	Vibration of functional groups	Primary compound class	Reference
3600−3000	3347	O–H groups (stretching vibration)	Alcohol	[34, 35]

3000−2500	2980	C–H stretching	Alkyne, alkene, alkane	[34, 35]

1670−1600	1640	C=C stretching	Alkene	[34, 35]

1600−1300	1417	Rocking vibrations of CH bond	Cis-disubstituted alkenes	[35]

1300−1000	1084	C–O stretching	Alcohol	[34]
	1043	S=O stretching	Sulfoxide	

1000−400	876	C–H bending	1,2,4-Trisubstituted or 1,3-disubstituted	[34]

**Table 2:** The average content of 21 elements in vodka from 5 brands (*μ*g/L).

	Aligator	Men's	Rocket	Vodka Hanoi	Hanoi Wine
^7^Li	1.05 ± 0.09	0.80 ± 0.07	0.81 ± 0.07	0.90 ± 0.08	0.47 ± 0.04
^23^Na	963 ± 97.57	1269.24 ± 98.95	205.67 ± 17.01	359.59 ± 31.46	886.92 ± 77.47
^24^Mg	267.49 ± 22.29	236.35 ± 17.42	65.57 ± 6.29	711.11 ± 68.38	1453.34 ± 127.7
^27^Al	93.69 ± 7.21	70.41 ± 6.37	49.2 ± 4.09	135.64 ± 11.98	28.16 ± 2.38
^39^K	636.54 ± 52.77	535.38 ± 45.22	239.33 ± 20.33	898.72 ± 79.31	5856.33 ± 482.39
^44^Ca	1052.01 ± 89.81	358.45 ± 32.99	255.72 ± 21.44	3157.16 ± 262.14	1337.39 ± 117.26
^48^Ti	5.62 ± 0.50	5.27 ± 0.44	3.4 ± 0.3	16.62 ± 1.44	4.41 ± 0.36
^51^V	0.70 ± 0.06	0.72 ± 0.06	0.61 ± 0.05	0.96 ± 0.08	1.26 ± 0.10
^52^Cr	14.18 ± 1.15	14 ± 1.26	23.2 ± 1.98	36.06 ± 3.14	<LOD^*∗*^
^55^Mn	18.12 ± 1.57	19.24 ± 1.66	3.01 ± 0.25	20.8 ± 1.59	22.76 ± 1.73
^57^Fe	232.83 ± 20.52	285.07 ± 22.93	121.52 ± 9.91	610.15 ± 49.34	31.9 ± 2.73
^59^Co	<LOD^*∗*^	3.9 ± 0.34	<LOD^*∗*^	<LOD^*∗*^	<LOD^*∗*^
^60^Ni	533.07 ± 44.16	81.77 ± 7.48	46.27 ± 4.52	1709.15 ± 147.71	2.06 ± 0.18
^63^Cu	5.2 ± 0.42	5.83 ± 0.56	6.23 ± 0.49	4.06 ± 0.35	5.82 ± 0.52
^66^Zn	933.46 ± 79.9	163.2 ± 14.12	38.66 ± 3.15	2771.85 ± 223.8	41.99 ± 3.92
^98^Mo	1.70 ± 0.14	1.62 ± 0.14	1.74 ± 0.15	2.93 ± 0.23	1.13 ± 0.1
^111^Cd	0.57 ± 0.05	0.49 ± 0.04	0.45 ± 0.04	0.71 ± 0.06	0.77 ± 0.07
^115^In	0.59 ± 0.05	0.59 ± 0.05	0.58 ± 0.05	0.59 ± 0.05	0.58 ± 0.05
^121^Sb	0.73 ± 0.06	0.18 ± 0.02	0.20 ± 0.02	2.05 ± 0.17	0.24 ± 0.02
^138^Ba	10.45 ± 0.94	7.66 ± 0.66	8.20 ± 0.81	7.90 ± 0.73	6.02 ± 0.51
^208^Pb	10.29 ± 0.91	10.26 ± 0.97	8.27 ± 0.79	11.47 ± 0.88	3.56 ± 0.27

^*∗*^LOD: limit of detection (3x standard deviation = 0.003 *μ*g/kg). Mean concentrations of elements in vodka samples (*μ*g/L) from different brands. Values represent the mean ± standard deviation of triplicate measurements for each of the 60 samples per brand. Triplicate measurements were conducted to ensure analytical robustness.

**Table 3:** FTIR Principal Component Analysis summary.

Component	*R* ^2^ *X*_(cumul.)_	*Q*_(cumul.)_^2^	Eigenvalue
1	0.465900	0.260397	7.454400
2	0.847812	0.760540	6.110598
3	0.883775	0.778942	0.575403
4	0.912802	0.792826	0.464426

**Table 4:** ICP-MS Principal Component Analysis summary.

Component	*R* ^2^ *X*_(cumul.)_	*Q*_(cumul.)_^2^	Eigenvalue
1	0.464806	0.409663	10.22573
2	0.730187	0.674752	5.83839
3	0.841583	0.777662	2.45070
4	0.897530	0.795026	1.23084
5	0.942537	0.836028	0.99015
6	0.956506	0.848622	0.30732
7	0.968163	0.868069	0.25645
8	0.975130	0.875074	0.15329

## Data Availability

The majority of the data used to support the findings of this study are included within the article. Other data are available from the corresponding author upon request.
